# Dissociable Networks of the Lateral/Medial Mammillary Body in the Human Brain

**DOI:** 10.3389/fnhum.2020.00228

**Published:** 2020-06-18

**Authors:** Masaki Tanaka, Takahiro Osada, Akitoshi Ogawa, Koji Kamagata, Shigeki Aoki, Seiki Konishi

**Affiliations:** ^1^Department of Neurophysiology, Juntendo University School of Medicine, Tokyo, Japan; ^2^Department of Radiology, Juntendo University School of Medicine, Tokyo, Japan; ^3^Research Institute for Diseases of Old Age, Juntendo University School of Medicine, Tokyo, Japan; ^4^Sportology Center, Juntendo University School of Medicine, Tokyo, Japan; ^5^Advanced Research Institute for Health Science, Juntendo University School of Medicine, Tokyo, Japan

**Keywords:** hippocampus, tegmentum, fornix, mammillotegmental tract, resting-state functional connectivity

## Abstract

The mammillary body (MB) has been thought to implement mnemonic functions. Although recent animal studies have revealed dissociable roles of the lateral and medial parts of the MB, the dissociable roles of the lateral/medial MB in the human brain is still unclear. Functional connectivity using resting-state functional magnetic resonance imaging (fMRI) provides a unique opportunity to noninvasively inspect the intricate functional organization of the human MB with a high degree of spatial resolution. The present study divided the human MB into lateral and medial parts and examined their functional connectivity with the hippocampal formation, tegmental nuclei, and anterior thalamus. The subiculum of the hippocampal formation was more strongly connected with the medial part than with the lateral part of the MB, whereas the pre/parasubiculum was more strongly connected with the lateral part than with the medial part of the MB. The dorsal tegmental nucleus was connected more strongly with the lateral part of the MB, whereas the ventral tegmental nucleus showed an opposite pattern. The anterior thalamus was connected more strongly with the medial part of the MB. These results confirm the extant animal literature on the lateral/medial MB and provide evidence on the parallel but dissociable systems involving the MB that ascribe mnemonic and spatial-navigation functions to the medial and lateral MBs, respectively.

## Introduction

The mammillary body (MB) receives inputs from the hippocampal formation and tegmental nuclei and sends outputs to the tegmental nuclei (Vann, 2010). Recent animal research has revealed that, while the lateral and medial MBs are connected to the same overall structures, they are connected to different subregions of these structures, thus forming two parallel but dissociable pathways (Vann and Aggleton, 2004): the medial MB receives inputs from the subiculum and ventral tegmental nucleus and projects to the ventral tegmental nucleus, while the lateral MB receives inputs from the pre/parasubiculum and dorsal tegmental nucleus and sends outputs to the dorsal tegmental nucleus (Vann, 2010; Dillingham et al., 2015). Functional dissociation between the lateral and medial MBs has also been demonstrated: behavioral studies of selective disconnections of the medial MB using discrete lesions of the mammillothalamic tract revealed that the medial MB is related to spatial memory (Vann and Aggleton, 2004), and electrophysiological studies reported that the lateral MB contains head-direction cells, indicating that the lateral MB contributes to spatial navigation (Blair et al., 1998; Stackman and Taube, 1998; Taube, 2007).

**Figure 1:**
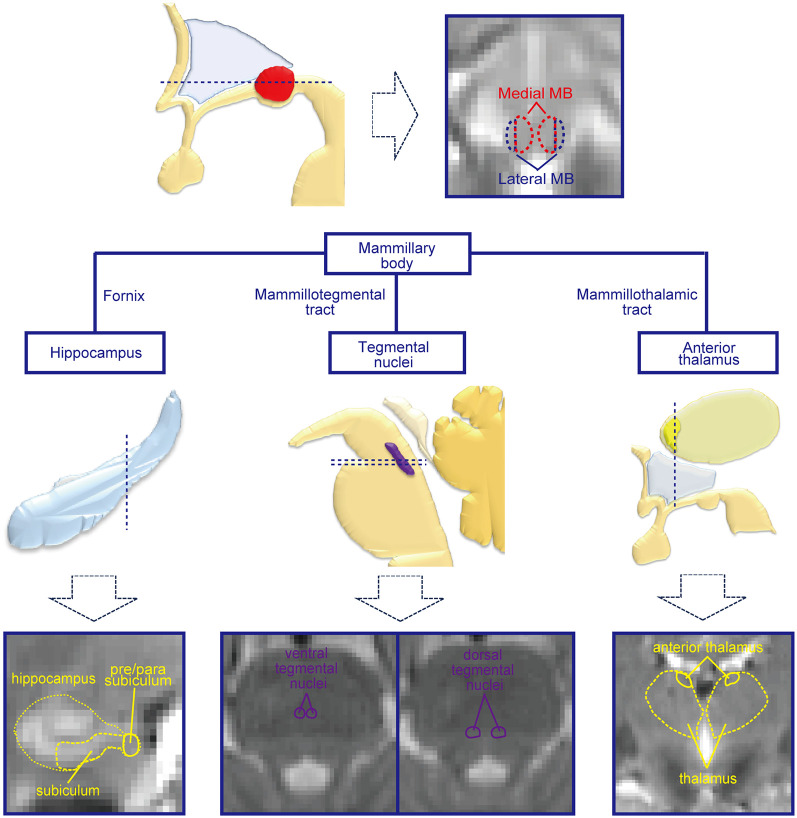
Mammillary body (MB) and regions connected with the MB. The MB receives inputs from the hippocampal formation and tegmental nuclei and sends outputs to the tegmental nuclei and anterior thalamus. By dividing the MB into lateral and medial parts, the functional connectivities with the hippocampal formation, tegmental nuclei, and anterior thalamus were investigated in the present study. Schematic drawings of the subcortical structures are shown in the middle row, and magnetic resonance imaging (MRI) images of the corresponding slices for the dashed lines are shown in the lowest row.

In humans, diffusion-weighted imaging studies revealed several tracts that connect with the MB, such as the fornix and mammillotegmental tract (Granziera et al., 2011; Kwon et al., 2011; Mori and Aggarwal, 2014; Christiansen et al., 2016; Cacciola et al., 2017; Wei et al., 2017; Kamali et al., 2018; Choi et al., 2019; Maller et al., 2019). Clinical studies also revealed that memory-impaired patients with Korsakoff syndrome exhibit atrophy of the MB (Squire et al., 1990; Harding et al., 2000; Tsivilis et al., 2008; Fama et al., 2012; Kril and Harper, 2012; Aggleton, 2014; Kopelman, 2015; Isenberg-Grzeda et al., 2016; Arts et al., 2017; Johnson and Fox, 2018). However, the lateral-medial dissociation in the MB is much less evident in humans than in animals. Functional connectivity analyses of resting-state data obtained with functional magnetic resonance imaging (fMRI) (Fox and Raichle, 2007; Honey et al., 2009; Biswal et al., 2010; Yeo et al., 2011; Margulies et al., 2016; Miyashita, 2016) may identify the lateral-medial dissociation in the human MB. Resting-state functional connectivity is known to reflect anatomical connectivity, and resting-state fMRI has been widely used to infer how strongly different brain areas are connected (Fox and Raichle, 2007). Notably, functional connectivity has been used in previous studies to demonstrate distinct compartments within the hippocampus that are connected with other brain structures (Poppenk and Moscovitch, 2011; Lacy and Stark, 2012; Libby et al., 2012; Duncan et al., 2014; Shah et al., 2018; Vos de Wael et al., 2018; Dalton et al., 2019) and small subcortical structures with differential functional connectivity profiles (Li et al., 2014; Hirose et al., 2016; Kline et al., 2016; Zhang et al., 2016, 2018; Kumar et al., 2017). The present study aimed to reveal the dissociation between the two systems consisting of the lateral/medial MB and target regions connected with the MB, such as the hippocampus and tegmental nuclei (Figure 1). The MB was divided into lateral and medial parts, and the resting-state functional connectivity was calculated. To attain a sufficient signal-to-noise ratio in functional images with a higher spatial resolution (1.25 × 1.25 × 1.25 mm^3^), each subject was highly sampled: 1,000 volumes in each of the 10 daily sessions.

## Materials and Methods

### Subjects

The present study reanalyzed the data published previously (Ogawa et al., 2018). Ten right-handed healthy subjects (six men and four women) participated in the experiments. The age of the subjects was in the category of young subjects [mean age, 27.0 ± 7.7 years (mean ± SD), age range, 20–39 years], as has most often been studied in normal subjects. They were confirmed to be healthy by annual medical checkups and had no psychiatric history. Written informed consent was obtained from all subjects according to the Declaration of Helsinki. All experimental procedures were approved by the Institutional Review Board of Juntendo University School of Medicine.

### MRI Procedures

fMRI data were acquired using a 3-T MRI scanner (Siemens Skyra, Erlangen, Germany). T1-weighted structural images were obtained for anatomical reference (resolution = 0.8 × 0.8 × 0.8 mm^3^). Functional images were obtained using multi-band gradient-echo echo-planar sequences [repetition time (TR) = 4.0 s, echo time (TE) = 41.6 ms, flip angle = 73°, field of view (FOV) = 160 × 160 mm^2^, matrix size = 128 × 128, 120 contiguous slices, voxel size = 1.25 × 1.25 × 1.25 mm^3^, multi-band factor = 4]. To attain a higher spatial resolution, a small FOV (160 × 160 mm^2^) was set (Osada et al., 2017; Ogawa et al., 2018). Although this small FOV did not always cover the posterior part of the occipital cortex, the areas of interest in the present study were intact. We acquired 100 volumes in each fMRI run at the resting state and repeated the process for 10 runs in each of the 10 daily sessions. Thus, 10,000 total volumes were collected for each subject.

### Image Analyses

Functional images were preprocessed for resting-state functional connectivity (Hirose et al., 2016; Osada et al., 2017, 2019; Ogawa et al., 2018; Tamura et al., 2019; Fujimoto et al., 2020). Images were corrected for slice timing and realigned using Statistical Parametric Mapping (SPM8) software^1^. Temporal filters (0.009 Hz < *f* < 0.08 Hz) were applied to the images using in-house-written MATLAB scripts. A general linear model was used to regress out nuisance signals that were correlated with head motion, whole-brain global signals, averaged ventricular signals, and averaged white matter signals. The obtained residual images were spatially normalized to the Montreal Neurological Institute (MNI) template with interpolation to a 1 × 1 × 1 mm^3^ space.

Then, we estimated the functional connectivity between the MB (the lateral/medial part of the MB or the whole MB) and target regions of interest (ROIs), such as the subiculum and pre/parasubiculum in the hippocampal formation, tegmental nuclei, and anterior thalamus, for each subject. Resting-state functional connectivities were calculated based on the procedures reported previously (Osada et al., 2017; Ogawa et al., 2018). The time-series signals in the lateral/medial part of the MB or whole MB were averaged across voxels (Figure 1). The averaged time-series signals in the seed MB regions were used to calculate their correlations with the time-series signals in the voxels of the target ROIs in the ipsilateral hemisphere. A voxel-wise correlation was calculated for the target ROIs, and the correlation coefficient was then converted to Fisher’s z and further to Gaussian *z* scores. The z score for each daily session was subject to the fixed effects model to obtain the z score for each subject. The resultant z-score images were spatially smoothed [full width at half maximum (FWHM) = 2 mm], and the z scores were averaged across voxels in the target ROIs for subsequent analyses of the lateral vs. medial part of the MB.

### Delineation of MB and Target ROIs

While the whole MB was manually delineated using normalized functional images, the lateral and medial MBs cannot be visually demarcated with MRI. The whole MB was covered by at most six parasagittal slices with a thickness of 1 mm. Because the volume of the medial MB was larger than that of the lateral MB (Vann, 2010; Corso et al., 2019), the whole MB was divided into lateral and medial parts by designating the two outer parasagittal slices as the lateral part of the MB and the four inner slices as the medial part of the MB. A 1-by-5 division of the slices was not adopted because every lateral slice may not have contained the lateral MB. The volumes of the lateral part of the MB were 28.4 ± 8.1 mm^3^ (mean ± SD) and 28.7 ± 9.0 mm^3^ in the left and right hemispheres, respectively. The volumes of the medial part of the MB were 89.3 ± 17.4 mm^3^ and 90.7 ± 10.4 mm^3^ in the left and right hemispheres, respectively.

The target ROIs consisted of the subiculum and pre/parasubiculum of the hippocampal formation, tegmental nuclei, and anterior thalamus; each was manually delineated using normalized functional and structural images. The subiculum and pre/parasubiculum were demarcated according to Yushkevich et al. (2015) and Dalton et al. (2017). Situated at the ventral part of the hippocampal formation, the subiculum and pre/parasubiculum were delineated based on the anatomical landmark of the vestigial hippocampal sulcus and uncal sulcus (Supplementary Figure S1A). The pre/parasubiculum is located medially to the subiculum. The borders between the two structures were delineated based on the histological atlas of the human hippocampus (Mai et al., 2007; Dalton et al., 2017) (Supplementary Figure S1B).

The ventral and dorsal tegmental nuclei were demarcated based on the histological atlas of the human brain stem (Paxinos and Mai, 2004; Naidich et al., 2009). The ventral tegmental nucleus is situated ventrally to the medial longitudinal fasciculus and extends approximately 4 mm rostrally from the level of the caudal pole of the locus coeruleus (Huang et al., 1992). The dorsal tegmental nucleus is located within the central gray matter and extends approximately 5 mm caudally from the level of the rostral pole of the locus coeruleus (Huang et al., 1992) (Supplementary Figure S1C).

The anterior thalamus consists of the anterior medial, anterior ventral, and anterior dorsal thalamic nuclei. Although the anterior ventral and anterior medial nuclei occupy most (approximately 90%) of the entire anterior nucleus (Kumar et al., 2017), the borders between the three structures are difficult to identify on MRI images. Performed according to the histological atlas of the human thalamus (Mai et al., 2007), our delineation was restricted to the entire anterior thalamus (Supplementary Figure S1D).

## Results

**Figure 2:**
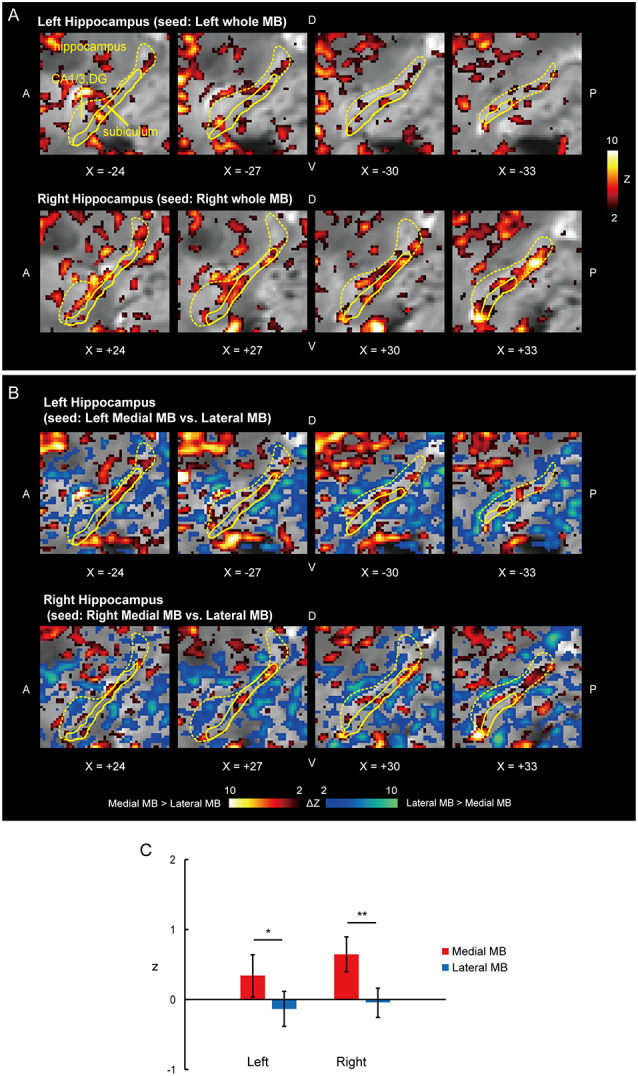
Functional connectivity in the subiculum. **(A)** Voxel-wise maps of functional connectivity in the ipsilateral hippocampus (seed: the whole MB) shown in the sagittal sections of functional images in one representative subject. The color scale indicates the Gaussian z score of the functional connectivity. The hippocampus and subiculum are delineated by yellow curves. X indicates the X coordinate of the Montreal Neurological Institute (MNI) space. A, anterior; P, posterior; D, dorsal; V, ventral. **(B)** Voxel-wise maps of differential functional connectivity in the hippocampus (seed: the medial vs. lateral MB). The color scale indicates the Gaussian z score of the differential functional connectivity (hot, medial > lateral; winter, lateral > medial). **(C)** Gaussian z score averaged across voxels in the left/right subiculum (seed: medial/lateral MB). The error bars indicate the standard error of means across subjects. *P < 0.05, **P < 0.01, paired *t*-test.

**Figure 3:**
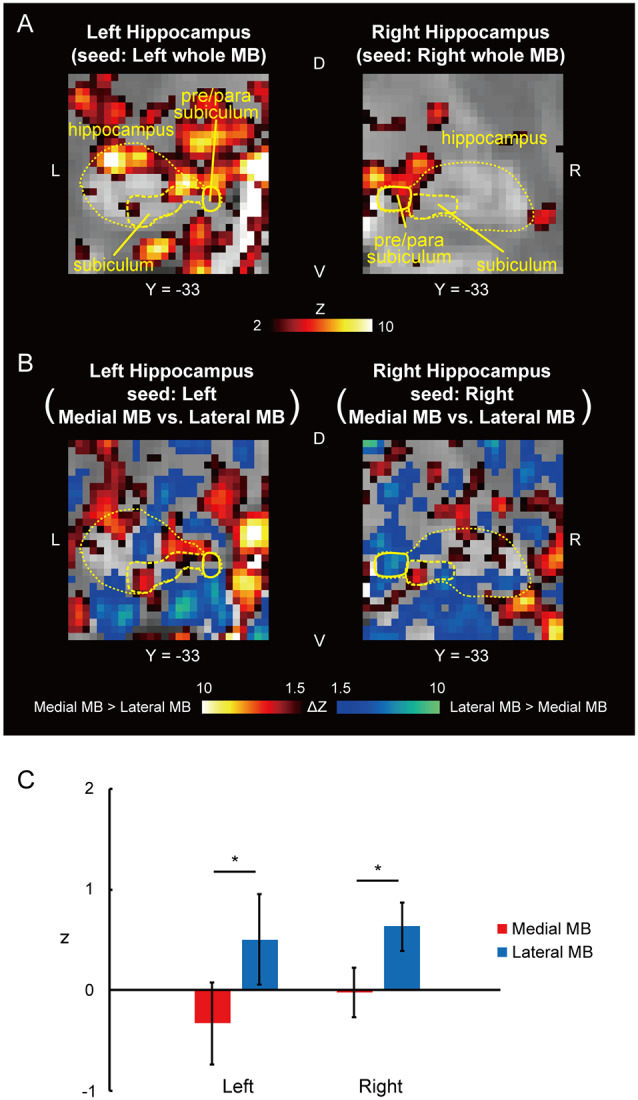
Functional connectivity in the pre/parasubiculum. **(A)** Voxel-wise maps of functional connectivity in the hippocampus (seed: the whole MB) shown in the coronal sections of one representative subject. The hippocampus, subiculum, and pre/parasubiculum are delineated by yellow curves. Y indicates the Y coordinate of the MNI space. L, left; R, right. **(B)** Voxel-wise maps of differential functional connectivity in the hippocampus (seed: the medial vs. lateral MB). **(C)** Gaussian z score averaged across voxels in the left/right pre/parasubiculum (seed: medial/lateral MB). *P < 0.05, paired *t*-test.

Functional connectivity was calculated between the whole MB or lateral/medial part of the MB and target ROIs in the subiculum, pre/parasubiculum, ventral/dorsal tegmental nucleus, and anterior thalamus. Figure 2A shows the functional connectivity between the whole MB and hippocampus in sagittal sections. The medial part of the MB featured greater connectivity (i.e., higher ROI-wise correlation values) with the subiculum than the lateral part of the MB (Figures 2B,C; see also Supplementary Figure S2A). Figure 3A shows the functional connectivity between the whole MB and hippocampus in coronal sections. The pre/parasubiculum was found to connect more strongly to the lateral part of the MB than to the medial part of the MB (Figures 3B,C; see also Supplementary Figure S2B). A three-way analysis of variance (ANOVA) was conducted with the region (subiculum or pre/parasubiculum), lateral/medial MB, and left/right sides as factors. A significant interaction (region × lateral/medial MB) was observed (*F*_(1,9)_ = 27.9, *P* = 0.0005; *P* = 0.002 after threefold Bonferroni correction for multiple comparisons), with no significant main effect (region, *F*_(1,9)_ = 0.002, *P* = 0.9; lateral/medial MB, *F*_(1,9)_ = 0.4, *P* = 0.5; left/right side, *F*_(1,9)_ = 0.7, *P* = 0.4). Since the hippocampus extends along the longitudinal axis, the subiculum was divided into three parts (Supplementary Figure S3), and a three-way ANOVA was performed with the lateral/medial MB, left/right subiculum, and position (anterior, middle, and posterior) as factors. We found a significant effect of the interaction between lateral/medial MB and position (*F*_(2,18)_ = 5.6, *P* = 0.01) but did not find a main effect of position (*F*_(2,18)_ = 1.1, *P* = 0.4).

**Figure 4:**
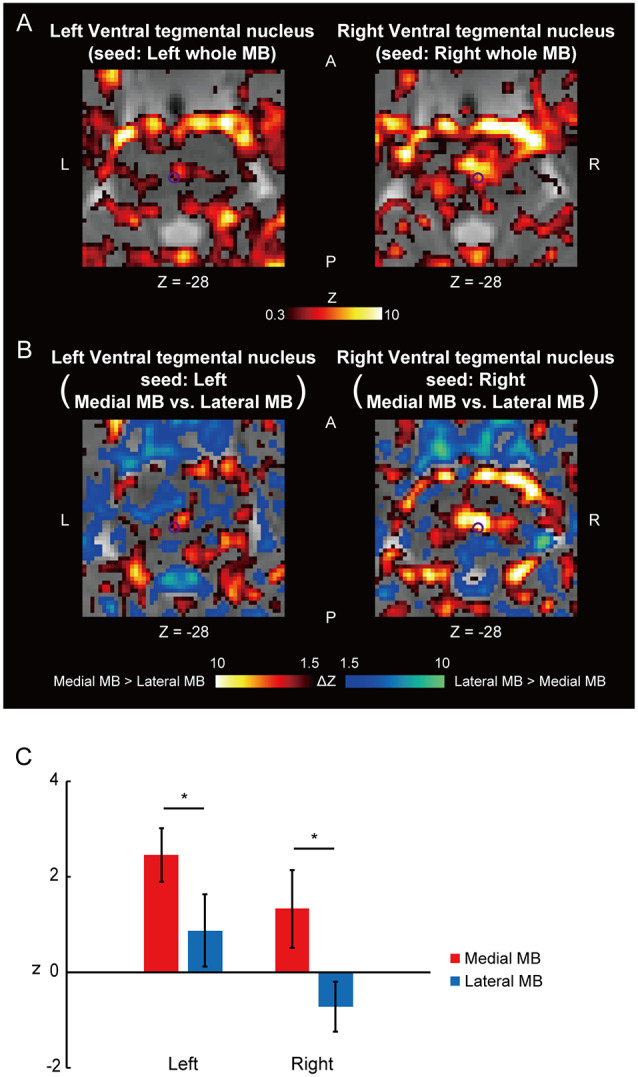
Functional connectivity in the ventral tegmental nucleus. **(A)** Voxel-wise maps of functional connectivity in the midbrain (seed: the whole MB) shown in the transverse sections of one representative subject. The ventral tegmental nucleus is delineated by a purple curve. Z indicates the Z coordinate of the MNI space. **(B)** Voxel-wise maps of differential functional connectivity in the midbrain (seed: the medial vs. lateral MB). **(C)** Gaussian z score averaged across voxels in the left/right ventral tegmental nucleus (seed: medial/lateral MB). *P < 0.05, paired *t*-test.

**Figure 5:**
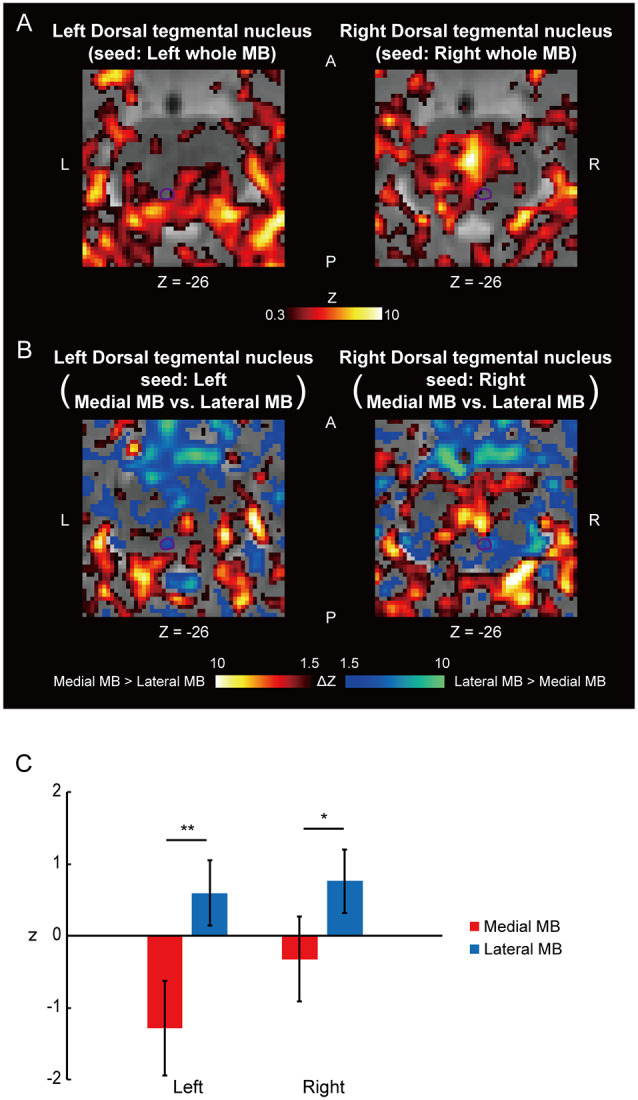
Functional connectivity in the dorsal tegmental nucleus. **(A)** Voxel-wise maps of functional connectivity in the midbrain (seed: the whole MB) shown in the transverse sections of one representative subject. The dorsal tegmental nucleus is delineated by a purple curve. **(B)** Voxel-wise maps of differential functional connectivity in the midbrain (seed: the medial vs. lateral MB). **(C)** Gaussian z score averaged across voxels in the left/right dorsal tegmental nucleus (seed: medial/lateral MB). *P < 0.05, **P < 0.01, paired *t*-test.

Figure 4A shows the functional connectivity between the whole MB and the ventral tegmental nucleus. The medial part of the MB exhibited greater connectivity with the ventral tegmental nucleus than the lateral part of the MB (Figures 4B,C; see also Supplementary Figure S2C). Figure 5A shows the functional connectivity between the whole MB and the dorsal tegmental nucleus. Differential functional connectivity was found: the lateral part of the MB was found to connect more strongly to the dorsal tegmental nucleus than the medial part of the MB (Figures 5B,C; see also Supplementary Figure S2D). A three-way ANOVA was conducted with the region (ventral or dorsal tegmental nucleus), lateral/medial MB, and left/right side as factors. A significant interaction (region × lateral/medial MB) was observed (*F*_(1,9)_ = 35.9, *P* = 0.0002; *P* = 0.0006 after threefold Bonferroni correction for multiple comparisons), with no significant main effect (region, *F*_(1,9)_ = 3.6, *P* = 0.09; lateral/medial MB, *F*_(1,9)_ = 0.3, *P* = 0.6; left/right side, *F*_(1,9)_ = 0.3, *P* = 0.6).

**Figure 6:**
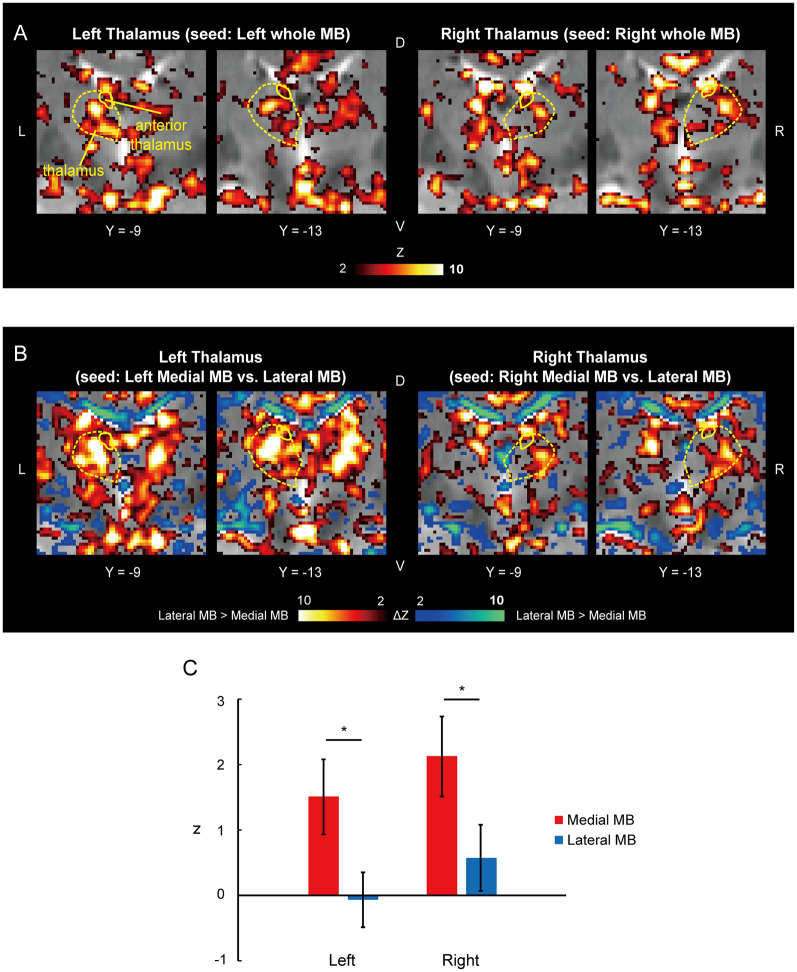
Functional connectivity in the anterior thalamus. **(A)** Voxel-wise maps of functional connectivity in the thalamus (seed: the whole MB) shown in the coronal sections of one representative subject. The whole thalamus and anterior thalamus are delineated by yellow curves. **(B)** Voxel-wise maps of differential functional connectivity in the thalamus (seed: the medial vs. lateral MB). **(C)** Gaussian z averaged across voxels in the left/right anterior thalamus (seed: medial/lateral MB). *P < 0.05, paired *t*-test.

Figure 6A shows the functional connectivity between the whole MB and thalamus. The medial part of the MB was found to exhibit stronger connections with the anterior thalamus than the lateral part of the MB (Figure 6B; see also Supplementary Figure S2E). A two-way ANOVA revealed a significant main effect of the lateral/medial MB (*F*_(1,9)_ = 16.3, *P* = 0.003; *P* = 0.009 after threefold Bonferroni correction for multiple comparisons) but no significant effect of the left/right anterior thalamus (*F*_(1,9)_ = 1.0, *P* = 0.3) or interaction between the two factors (*F*_(1,9)_ = 0.002, *P* = 0.9; Figure 6C). Furthermore, no significant correlations were found between whole MB-subiculum and whole MB-anterior thalamus connectivities (*r* = 0.1, *P* = 0.5) or between whole MB-pre/parasubiculum and whole MB-anterior thalamus connectivities (*r* = 0.2, *P* = 0.4).

The power analysis was conducted to estimate the minimum sample size necessary for achieving 80% power at an alpha of 0.05 for the main/interaction effects in the ANOVA. The minimum sample size was 6, 5, and 7 in the hippocampal formation, tegmental nuclei, and anterior thalamus, respectively. The power analysis confirmed that the sample size in the present study satisfied the requirement.

## Discussion

The present fMRI study investigated the two pathways involving in the human lateral and medial MBs by using resting-state functional connectivity. The medial MB was functionally connected with the subiculum, ventral tegmental nucleus, and anterior thalamus, whereas the lateral MB was functionally connected with the pre/parasubiculum and dorsal tegmental nucleus. Previous animal studies have shown that the medial MB is involved in memory functions (Kirk et al., 1996; Vann and Aggleton, 2004; Sharp and Turner-Williams, 2005; Vann, 2010); and the lateral MB, in spatial navigation (Blair et al., 1998; Stackman and Taube, 1998; Vann, 2005; Taube, 2007; Harland et al., 2017). The present study validates previous evidence provided by animal studies for the parallel but dissociable systems comprising in human MBs as well as the updated Papez circuit (Vann and Nelson, 2015) in the human brain, which was revised to include the dorsal and ventral tegmental nuclei.

It is known that the medial MB occupies the majority of the volume of the whole MB (Vann, 2010; Corso et al., 2019). Out of the six slices that covered the whole MB, two lateral slices were designated as the lateral part of the MB in the present study. Designating only the most lateral slice as the lateral part of the MB (1-by-5 division) will not be optimal because the one slice may not have contained the lateral MB. A 3-by-3 division was not adopted because the volume of the medial MB is known to be much larger than that of the lateral MB. Therefore, we adopted the 2-by-4 division. It is also possible, on the other hand, that the two lateral slices contained the medial MB. However, the results of the pre/parasubiculum and dorsal tegmental nucleus showing the lateral-dominant connectivity pattern (Figures 3, 5) confirmed the lateral vs. medial dissociation in the MB.

The classical Papez circuit consists of the hippocampal formation, MBs, anterior thalamus, and cingulate cortex (Papez, 1937). Recent studies have proposed an updated conception of the Papez circuit (Vann and Nelson, 2015), which includes the dorsal and ventral tegmental nuclei. Animal studies have found that the dorsal and ventral tegmental nuclei project to the lateral and medial MBs, respectively, to form two parallel but dissociable pathways (Vann, 2010; Saunders et al., 2012; Dillingham et al., 2015). It has also been demonstrated that lesions in the ventral tegmental nucleus cause memory impairments (Vann, 2010), whereas lesions in the dorsal tegmental nucleus induce loss of head-direction cells in the lateral MB (Bassett et al., 2007). The present study provides the human analog of the two extended systems that implement different functions whose underlying biological mechanism is centered in the lateral and medial MBs.

## Data Availability Statement

The datasets generated for this study are available on request to the corresponding author.

## Ethics Statement

The studies involving human participants were reviewed and approved by Institutional Review Board of Juntendo University School of Medicine. The patients/participants provided their written informed consent to participate in this study.

## Author Contributions

MT, TO, and SK designed the study, collected the data, analyzed the data and drafted the article. AO, KK, and SA collected the data and drafted the article.

## Conflict of Interest

The authors declare that the research was conducted in the absence of any commercial or financial relationships that could be construed as a potential conflict of interest.

## Footnotes

^1^https://www.fil.ion.ucl.ac.uk/spm/

## Supplementary Material

FIGURE S1 Target regions of interest (ROIs) on functional images. **(A,B)** The hippocampus, subiculum, and pre/parasubiculum are delineated by yellow curves in the sagittal **(A)** and coronal **(B)** sections of functional images in one representative subject. X indicates the X coordinate of the Montreal Neurological Institute (MNI) space. Y indicates the Y coordinate of the MNI space. US, uncal sulcus; VHS, vestigial hippocampal sulcus; A, anterior; P, posterior; D, dorsal; V, ventral; L, left; R, right. **(C)** The ventral and dorsal tegmental nuclei are delineated by purple curves in the transverse sections of one representative subject. The medial longitudinal fasciculus and central gray matter are delineated by white curves. Z indicates the Z coordinate of the MNI space. **(D)** The whole thalamus and anterior thalamus are delineated by yellow curves in the coronal section of one representative subject. The body of fornix is delineated by a white curve.

FIGURE S2 Differential functional connectivity maps. **(A)** Voxel-wise maps of differential functional connectivity in the hippocampus (seed: the medial vs. lateral mammillary body (MB)). The color scale indicates the Gaussian z of the differential functional connectivity (hot: medial > lateral, winter: lateral > medial). Differential functional connectivity maps were shown only within the hippocampus for display purposes. **(B)** Voxel-wise maps of differential functional connectivity in the hippocampus (seed: the medial vs. lateral MB). Differential functional connectivity maps were shown only within the hippocampus for display purposes. **(C)** Voxel-wise maps of differential functional connectivity in the midbrain (seed: the medial vs. lateral MB). Differential functional connectivity maps were shown only within the midbrain for display purposes. Z indicates the Z coordinate of the MNI space. **(D)** Voxel-wise maps of differential functional connectivity in the midbrain (seed: the medial vs. lateral MB). Differential functional connectivity maps were shown only within the midbrain for display purposes. **(E)** Voxel-wise maps of differential functional connectivity in the thalamus (seed: the medial vs. lateral MB). Differential functional connectivity maps were shown only within the thalamus for display purposes.

FIGURE S3 Functional connectivity in the three subdivisions of the subiculum. Gaussian z averaged across voxels in the three subdivisions (anterior, middle, and posterior) of the subiculum (seed: medial/lateral MB). The three subdivisions are delineated by colored curves in the sagittal section. **P* < 0.05, ***P* < 0.01, paired *t*-test.
