# Supplementary figures and images for: Dissociable Networks of the Lateral/Medial Mammillary Body in the Human Brain
Source: Front Hum Neurosci. 2020 Jun 18;14:228. doi: 10.3389/fnhum.2020.00228 (PMC7316159; doi:10.3389/fnhum.2020.00228)

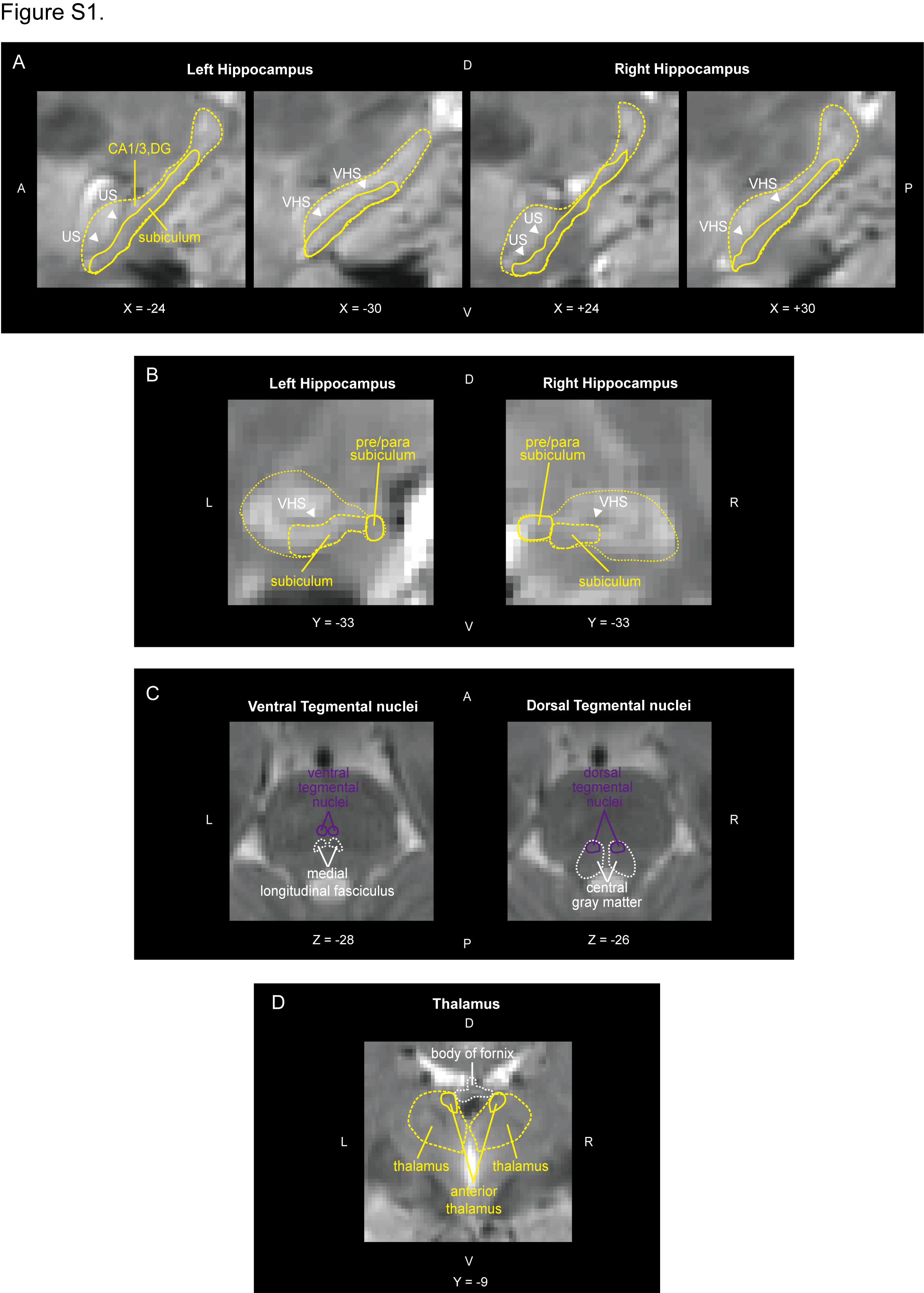

Supplement: FIGURE S1 — Target regions of interest (ROIs) on functional images. (A,B) The hippocampus, subiculum, and pre/parasubiculum are delineated by yellow curves in the sagittal (A) and coronal (B) sections of functional images in one representative subject. X indicates the X coordinate of the Montreal Neurological Institute (MNI) space. Y indicates the Y coordinate of the MNI space. US, uncal sulcus; VHS, vestigial hippocampal sulcus; A, anterior; P, posterior; D, dorsal; V, ventral; L, left; R, right. (C) The ventral and dorsal tegmental nuclei are delineated by purple curves in the transverse sections of one representative subject. The medial longitudinal fasciculus and central gray matter are delineated by white curves. Z indicates the Z coordinate of the MNI space. (D) The whole thalamus and anterior thalamus are delineated by yellow curves in the coronal section of one representative subject. The body of fornix is delineated by a white curve. [file Image_1.TIF]

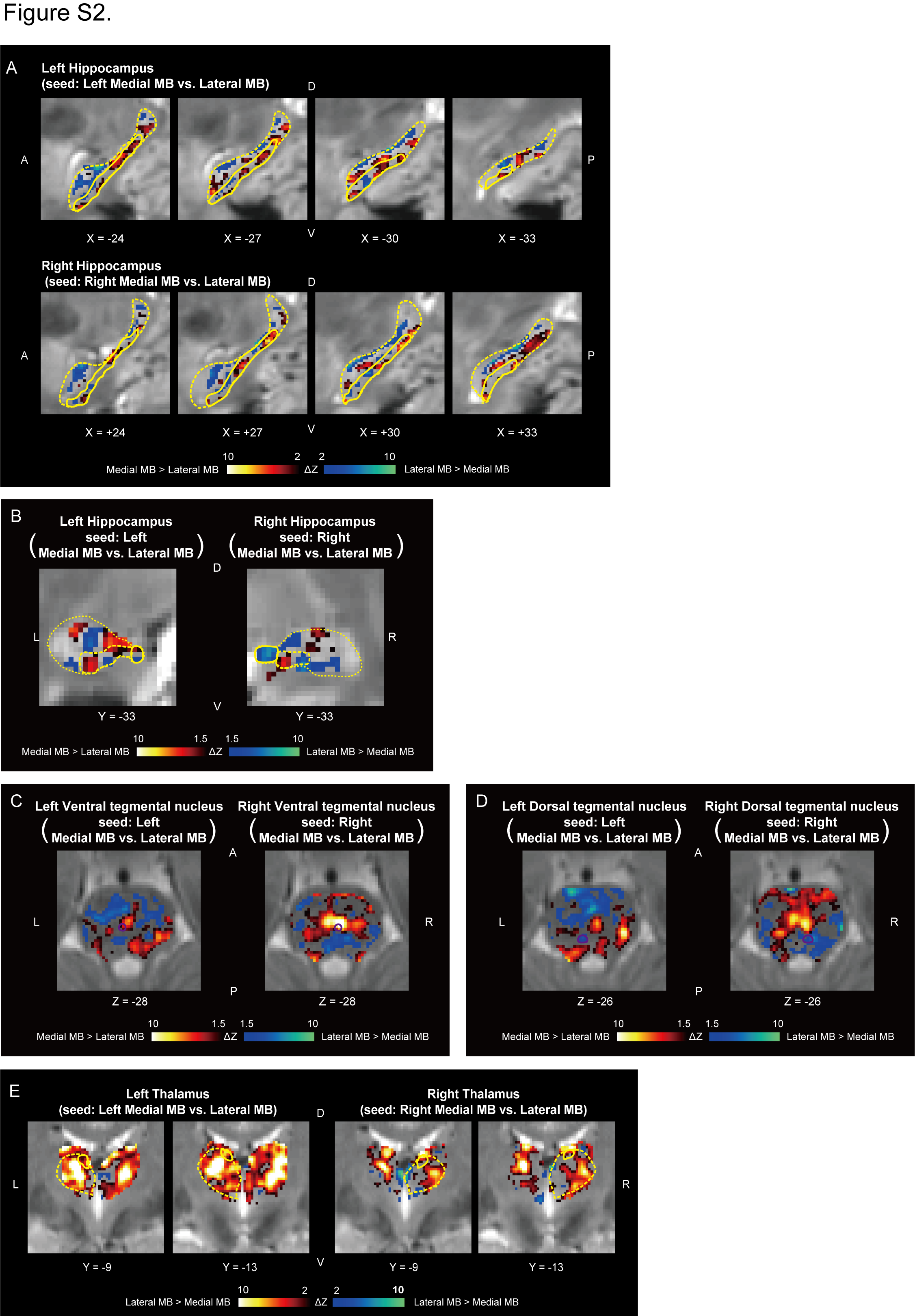

Supplement: FIGURE S2 — Differential functional connectivity maps. (A) Voxel-wise maps of differential functional connectivity in the hippocampus (seed: the medial vs. lateral mammillary body (MB)). The color scale indicates the Gaussian z of the differential functional connectivity (hot: medial > lateral, winter: lateral > medial). Differential functional connectivity maps were shown only within the hippocampus for display purposes. (B) Voxel-wise maps of differential functional connectivity in the hippocampus (seed: the medial vs. lateral MB). Differential functional connectivity maps were shown only within the hippocampus for display purposes. (C) Voxel-wise maps of differential functional connectivity in the midbrain (seed: the medial vs. lateral MB). Differential functional connectivity maps were shown only within the midbrain for display purposes. Z indicates the Z coordinate of the MNI space. (D) Voxel-wise maps of differential functional connectivity in the midbrain (seed: the medial vs. lateral MB). Differential functional connectivity maps were shown only within the midbrain for display purposes. (E) Voxel-wise maps of differential functional connectivity in the thalamus (seed: the medial vs. lateral MB). Differential functional connectivity maps were shown only within the thalamus for display purposes. [file Image_2.TIF]

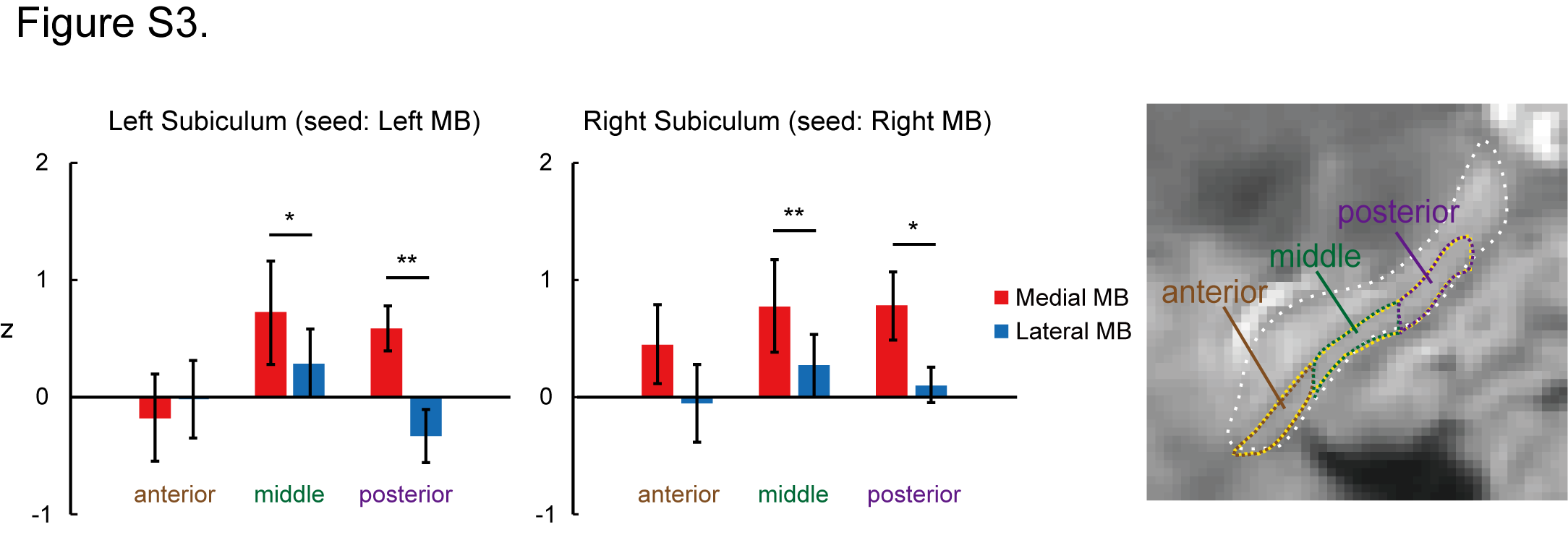

Supplement: FIGURE S3 — Functional connectivity in the three subdivisions of the subiculum. Gaussian z averaged across voxels in the three subdivisions (anterior, middle, and posterior) of the subiculum (seed: medial/lateral MB). The three subdivisions are delineated by colored curves in the sagittal section. *P < 0.05, **P < 0.01, paired t-test. [file Image_3.TIF]
